# Patient Knowledge about Oral Anticoagulation Therapy Assessed during an Intermediate Medication Review in Swiss Community Pharmacies

**DOI:** 10.3390/pharmacy8020054

**Published:** 2020-03-28

**Authors:** Corina Metaxas, Valerie Albert, Susanne Habegger, Markus Messerli, Kurt E. Hersberger, Isabelle Arnet

**Affiliations:** Department of Pharmaceutical Sciences, Pharmaceutical Care Research Group, University of Basel, Petersplatz 14, Postfach 2148, 4001 Basel, Switzerland; valerie.albert@unibas.ch (V.A.); s_habegger@bluewin.ch (S.H.); markus.messerli@unibas.ch (M.M.); kurt.hersberger@unibas.ch (K.E.H.); isabelle.arnet@unibas.ch (I.A.)

**Keywords:** oral anticoagulation, patient medication knowledge, medication review

## Abstract

**Background:** Therapy with oral anticoagulation (OAC) can be challenging, especially in high risk groups such as chronic patients. Gaps in patient knowledge about OAC are linked to reduced effectiveness and safety of treatment. The objectives of this study were i) to assess OAC knowledge gathered during an intermediate medication review (MR) in patients taking vitamin K antagonists (VKA) or non-vitamin K antagonist oral anticoagulants (NOAC); ii) to assess OAC knowledge two weeks after the MR, and iii) to evaluate patient satisfaction with the MR service in community pharmacies. **Methods:** Chronic OAC patients were invited for a regular MR service in Swiss community pharmacies, the so-called “Polymedication-Check” (PMC). OAC knowledge was assessed with seven newly generated items asked face-to-face during a PMC and by telephone two weeks later. Knowledge gaps, pharmacists’ spontaneous interventions, and patient satisfaction were documented by observing pharmacy students. Treatment groups were compared. **Results:** Of all patients (n = 81), the number of patients with one or more knowledge gaps decreased from 66% to 31.3% after PMC (*p* < 0.001). NOAC patients (n = 31) had more knowledge gaps than VKA patients (n = 50; *p* < 0.05). Most patients (98.6%) were satisfied with the counselling provided by the pharmacists. **Conclusion:** The majority of chronic OAC patients shows knowledge gaps. Although spontaneous, the provision of tailored education during a PMC increased patient OAC knowledge.

## 1. Introduction

Oral anticoagulation (OAC) is prescribed for the prevention and therapy of thromboembolic diseases [1,2,3] with either vitamin K antagonists (VKA) or non-vitamin K antagonist oral anticoagulants (NOAC), also called direct oral anticoagulants. The chronic nature of anticoagulant treatment and the inherent bleeding risk increase the challenge of treatment for both types of OAC. NOACs were developed predominantly to overcome the practical difficulties associated with VKAs (i.e., frequent dosage adjustments, drug-drug and drug-food interactions, among others) [4]. However, strict intake time of VKA and NOAC, that is high medication adherence, is needed to achieve optimal therapeutic outcome [5]. Insufficient knowledge about safety relevant aspects of OAC is likely to be a reason for therapeutic complications [6]. Gaps in patient knowledge were found to be one of the greatest barriers toward OAC in patient focus groups [7] and were associated with reduced safety and effectiveness of OAC [6]. Overall safety-relevant knowledge about OAC was lower in high risk groups such as older adults [8]. Moreover, several studies showed that insufficient patient knowledge about OAC was related to poor adherence [9,10] and inadequate anticoagulation control [8,11].

According to a Cochrane review, there is insufficient evidence to draw firm conclusion regarding the impact of educational interventions on therapeutic outcomes in patients receiving OAC [12] mostly due to the inhomogeneity of study designs. However, few studies showed that enhancing patient knowledge about OAC and the underlying disease was able to improve long-term adherence [13,14]. In addition, pharmacist-led verbal interventions on anticoagulation management were able to improve anticoagulation control in patients taking VKA [15,16]. A recent study on educational counselling by pharmacists was able to improve and maintain knowledge about OAC in a clinical setting [17]. Thus, educational counselling by pharmacists might improve patient knowledge about OAC, which might influence adherence and ultimately therapeutic outcome.

Simple screening tools could be useful for the detection of knowledge gaps. However, interventions aimed at increasing medication knowledge are likely to succeed if the entire medication i.e., polypharmacy, is taken into account, especially in older adults. Therefore, starting with a medication review (MR) [18] might be indispensable when patients are to be counselled about knowledge or adherence [19]. 

The Polymedication-Check (PMC) was introduced in Switzerland in 2010 as an intermediate MR for primary care [19]. The PMC is a cognitive service and is based on the Medicines Use Review (MUR) from the United Kingdom [20]. Comparable pharmacy-led services are available in several other countries such as Australia, Canada, the United States of America, the United Kingdom, and New Zealand [21,22,23,24]. The PMC allows the community pharmacist to address adherence issues, drug-related problems, and to provide direct interventions such as the implementation of weekly pill organizer [19]. The PMC is conducted with patient consent independently from the prescriber.

Usually, community pharmacists are the last healthcare providers (HCP) seen by patients before they take their medication. In Switzerland, physicians hand out prescriptions for chronic diseases that are valid for up to one year. Therefore, pharmacists are in a central position to assess patient degree of medication knowledge, to counsel, and to provide a follow-up, beside the delivery of the medication. The ability of detecting knowledge gaps in patients with chronic treatments such as OAC could help pharmacists or other HCPs to enhance adherence and safety of treatment.

We amended the basic PMC with seven open-ended knowledge questions about OAC. We aimed at evaluating patient baseline OAC knowledge and comparing it to OAC knowledge obtained after the MR, which encompasses unstructured interventions given by pharmacists. We explored differences between VKA and NOAC patients. Patient satisfaction with the service was evaluated.

## 2. Materials and Methods 

### 2.1. Trial Design

A cross-sectional study was initiated by the Pharmaceutical Care Research Group (PCRG) of the University of Basel. Swiss community pharmacies that employed pharmacy students in their final year (internship) served as recruiting places. The study was conducted in accordance with the Declaration of Helsinki and has been registered at ClinicalTrials.gov ID NCT02703727. The study was approved by the regional ethic committee (EKNZ 50/12). All patients gave written informed consent.

### 2.2. Data Collection

Fifth year pharmacy students were trained on OAC and PMC during plenary lectures of the regular curriculum at the University of Basel. A descriptive manual and an online training including a video, which describes the study procedure and provides instructions for the correct use of the amended PMC and the follow-up interview, were available for the students and the pharmacists. 

Community pharmacists were instructed to perform a basic PMC with one orally anticoagulated patient in the pharmacy and to seamlessly continue with the seven-item semi-structured interview (Part A). Students observed silently the patient-pharmacist encounter. They used a standardized report form to document all pharmacist-led interventions. Patient characteristics were obtained through filling in a questionnaire (age, gender, duration of OAC therapy). Two weeks later, students called the patients by phone and performed a follow-up interview (Part B).

#### 2.2.1. Basic Polymedication-Check (PMC) 

The basic PMC contains several items to be checked for each single medication ([19] in supplementary data). According to patient response, the pharmacist ticks the appropriate box in the PMC report form. After gathering information, the pharmacist can deliver tailored information in an unstructured manner [25]. OAC agent and co-medication were extracted from PMC. Other data of the basic PMC were not evaluated in this study.

#### 2.2.2. Development of the Amended PMC (Part A)

Thirteen items from a French publication about the evaluation of a pharmaceutical interview in an anticoagulation clinic [26] were translated into German by a German native speaker (CM). Readability and comprehension were approved by three German native speaking pharmacists (MM, SH, KH). Five items were excluded because they were not specific for OAC (n = 2) or did not concern knowledge about OAC (n = 3). The remaining eight items delineating knowledge were formulated as open-ended questions (Table 1). To each question corresponds one individual correct answer (Example: How do you take your OAC? Twice daily). Asking these eight questions in form of a semi-structured interview was supposed to take about 10 min.

#### 2.2.3. Development of the Follow-up Interview (Part B)

Five items about patient satisfaction were retrieved from a prior study [27] (Table 1). Answers were given on a 5-point-Likert scale with options ranging from 1 (=‘I do not agree’) to 5 (=‘I do agree’). The option ‘no answer’ was also available. The eight questions delineating knowledge in the amended PMC (part A) completed the follow-up interview and were asked first.

#### 2.2.4. Cognitive Testing of the Instrument

A total of 73 fifth year pharmacy students of the prior academic year tested the comprehension of the eight items to assess OAC knowledge and the five items to assess satisfaction; practicability of the standardized protocol to document patient demographics, pharmacist interventions, answers to follow-up telephone interviewand feasibility of conducting the study. The knowledge question asking for the name of the OAC was judged inadequate in light of the running of the PMC, as the pharmacist starts the encounter with explicitly naming the OAC product. The question was deleted. Minor wording adaptions were made.

### 2.3. Eligibility for patients

Patients were eligible if they fulfilled the official selection criteria for a basic PMC (age ≥18 years, ≥4 prescribed medications for ≥3 months [20]), if they were taking a VKA (acenocoumarol, phenprocoumon) or a NOAC (apixaban, dabigatran, edoxaban, rivaroxaban) not for orthopaedic indication, and accepted to sign the informed consent form.

### 2.4. Outcome Variables

Negative or incorrect answers to the seven knowledge questions were defined as “knowledge gaps” (items 1–7) and summed up. Satisfaction was obtained from a 5-point Likert scale with option for “no answer”. Pharmacist-led interventions were retrieved from the standardized protocols filled in by students.

### 2.5. Statistical Methods 

Where appropriate, mean and standard deviations or median and interquartile ranges are presented. The Mann-Whitney-U-test was used to compare numerical data and Chi-Square-test to compare categorical data between groups. McNemar’s test was used to compare paired proportions before and after the amended PMC. Comparison of data before and after the amended PMC was restricted to patients with full datasets. Data were entered and analyzed using SPSS statistical package version 26.0 (SPSS, Inc., Chicago, Illinois, USA) and *p*-values <0.05 were considered significant.

## 3. Results

### 3.1. Participants 

A total of 91 students collected data during their internship between November 2015 and June 2016. Two pharmacies declined participation and eight pharmacies failed to recruit a patient with OAC during the study period. 

Out of the 81 recruited patients, five (6.2%) were lost in follow-up (Table 2). Phenprocoumon was the most common VKA prescribed (phenprocoumon: 94%, n = 47; acenocoumarol: 6%, n = 3). Out of the 31 NOAC patients, 28 (90.3%) received rivaroxaban, 2 (6.5%) dabigatran, and one (3.2%) apixaban. Age, gender, and number of prescribed medication were equally distributed between patients treated with VKA or with NOAC (Table 2). The preceding duration of treatment was significantly longer for patients taking VKA than NOAC (*p* < 0.001; Table 2). 

### 3.2. Time

Average time needed to perform the amended PMC and the follow-up interview was 36 ± 15 min and 17 ± 12 min, respectively, and did not differ between VKA and NOAC patients (data not shown). Average time until follow-up was 15 ± 9 days.

### 3.3. Knowledge Gaps

#### 3.3.1. Additional Questions Amending the PMC (Part A)

To the 6 patients who answered incorrectly to item 1 (“knows how”) and to the 9 patients who answered incorrectly item 2 (“knows why”), pharmacists delivered an immediate intervention in 28.3% and 42.9% of the cases, respectively. “Underdosage” (item 3) and “overdosage” (item 4) were both not known by 44 patients (27.2%; Table 3). The majority of patients (63.0%, n = 51) ignored how to proceed in case of a missed dose (item 5; Table 3). “Treatment period” (item 6) and “HCP information” (item 7) were unknown for 8 (9.9%) and 18 (22.2%) patients, respectively. Identification of knowledge gaps in items 3–7 were followed by an immediate intervention in 28.6–90.0% of the cases. 

NOAC patients had significantly more knowledge gaps than VKA patients (NOAC: 2(1/3); VKA: 1(0/2); *p* < 0.05). NOAC patients ignored significantly more often how to take their medication (item 1; *p* < 0.01) and the consequences of overdosage (item 4; *p* < 0.05) compared to VKA patients. 

#### 3.3.2. Follow-up Interview (Part B)

Missing data reached 1.5%. The percentage of patients with knowledge gaps observed during the amended PMC for the questions “knows why”, “underdosage”, “missed dose” and “HCP information” (items 2, 3, 5, and 7) decreased significantly during the follow-up interview (Table 3). For the “treatment period” (item 6), the percentage of patients with knowledge gaps increased marginally at follow-up (Table 3). The percentage of patients with one or more knowledge gaps during the amended PMC decreased significantly from 66.0% to 31.3% at follow-up (*p* < 0.001).

### 3.4. Patient Satisfaction 

**Figure 1:**
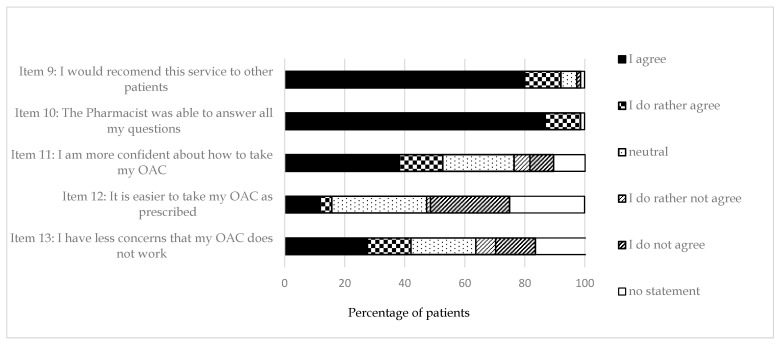
Answers from 81 patients to five close-ended questions on satisfaction with the amended PMC assessed during the follow-up telephone interview (Part B).

The majority of the patients would recommend the amended PMC to other people (92.0%), was satisfied with the answers given by the pharmacists (98.6%), and stated to be more confident with how to take the OAC after receiving the amended PMC (52.7%). More patients agreed that they had less concerns about their OAC after the amended PMC (42.1%) than were neutral (27.6%) or disagreed (30.3%; Figure 1). 

## 4. Discussion

In our study, 66% of the patients had at least one knowledge gap concerning their therapy with OAC during a semi-structured interview. Patients taking NOAC had overall more knowledge gaps compared to VKA patients. A spontaneous pharmacist-led counselling after a medication review was able to significantly reduce knowledge gaps. Patients were satisfied with the counselling that amended the service.

Our findings of inadequate OAC knowledge in chronic patients during a medication review such as the PMC (Part A) is in agreement with results of other studies that investigated patient knowledge about OAC [6,28,29]. 

Surprisingly, the majority of patients showed knowledge gaps when asked “What to do in case of a missed dose” (item 6), while around one-quarter of patients ignored what happens in case of underdosage or overdosage. A study investigating patient knowledge about warfarin found also that only half of the patients or fewer knew what happens in case of a missed dose, overdosage, or underdosage [30]. 

We further observed that patients taking NOAC were more likely to have knowledge gaps compared to patients taking VKA. The knowledge gaps of patients under NOAC therapy concerned mostly the questions ‘How to take the medication’ and ‘What happens in case of overdosage’ Compared to patients with VKA therapy, the number of knowledge gaps concerning these two questions was higher. These observations are in line with findings of a recent study [31]. Patients taking VKA had higher knowledge scores compared to patients taking NOAC. One reason might be the duration of therapy, which was significantly shorter in NOAC patients compared to VKA patients, suggesting that contact of NOAC patients with HCPs and possible education was limited. Another reason may be that routine blood testing in VKA patients allows self-reflection on medication intake behavior and thereby increases awareness of VKA therapy. 

Our results suggest that patient degree of OAC knowledge leaves something to be desired in Switzerland. However, a study performed in elderly patients with polypharmacy showed that patients overestimate their knowledge about therapy [27]. Therefore, knowledge assessments should be integrated in counselling sessions and offered periodically in patients taking VKA or NOAC.

Observational studies demonstrated that community pharmacists provide little medication-related information at the counter [32] and about half of the patients do not receive any counselling [25]. Contrary to these findings, we found that detection of poor knowledge through an amended PMC triggered pharmacists to provide unstructured but targeted patient education and thereby significantly reduced knowledge gaps at follow-up. 

Interestingly, 23.8% of patients remained with knowledge gaps after counselling. One reason might be that counselling session with the amended PMC took a mean of 45 ± 20 min, which is about 15 min longer than the duration of the basic PMC (29.8 ± 16.5 min [19]). Training of pharmacists might reduce the time needed to perform an amended PMC and increase intervention rates. Furthermore, training for pharmacists to incorporate patient-centered type of communication might trigger successful medication intake behavior in patients [33]. However, the duration of the amended PMC might be a barrier for its implementation in practice, if not better remunerated than the basic PMC. Additionally, the length of a counselling session may be critical in practice, as the advanced age of our population (72.5 ± 12.3 years) might go along with reduced cognitive capacity leading to loss of concentration. Thus, it may be reasonable to amend the PMC with specific questions only for high-risk medication such as OAC. We therefore suggest to further adapting the PMC to a specific anticoagulation MR, similar to the medicines use review in UK, which was also adapted for specific target groups [34]. Two studies reported on the use of video training for educational counselling on VKA [35,36]. Video education was shown to significantly decrease visit time, while comprehension or patient satisfaction remained stable [36]. Whether the results can be transferred to older chronically ill adults taking NOAC should be addressed in further studies. 

Within our study, we did not collect data on possible reasons for incorrect answers at follow-up. For further studies, it might be helpful to assess baseline memory function and health literacy in order to understand learning abilities of patients and to make comparison between different populations. The mean time interval between the amended PMC and the follow-up interview was 15 ± 9 days. Recommendations for interval between two identical tests vary between two days [37] and three months [38]. We selected a short time interval based on ethical considerations, because patients with deficient knowledge should be corrected as soon as possible in order to avoid life-threatening situations. Long-term sustainability of the increased knowledge about OAC and subsequent influence on behavior such as adherence and safe medicines use need to be addressed in randomized controlled trials.

We acknowledge some limitations. First, the presence of students as silent observers during a counselling encounter could have triggered community pharmacists to engage more in counselling practice than usual. However, this Hawthorne effect [39] was probably marginal as pharmacists are trained to mentoring students during internship. Second, the amended PMC included seven additional questions, which do not cover all educational domains important for OAC according to Wofford [40]. However, because this study was not VKA specific, topics such as blood-testing and food-drug interactions were omitted on purpose. In further studies, different sets of questions for either NOAC or VKA might be adequate. Third, an increased knowledge might have occurred at follow-up because the study may have increased patient awareness of OAC therapy. 

## 5. Conclusions

In our study, the majority of anticoagulated patients showed knowledge gaps concerning their therapy with OAC. Detection of deficient knowledge through the amended PMC triggered community pharmacists to provide spontaneous educational counselling, which in turn increased patient OAC knowledge. Further, patients showed high acceptance of the service. However, adaptions regarding duration and structure of the counselling encounter and specific screening questions for patients with VKA or NOAC might be helpful before implementing this new pharmacy service in practice. 

## Figures and Tables

**Table 1:** Content of the seven open-ended questions added to the basic Polymedication-Check (PMC) and forming the semi-structured interview (Part A) and the additional five close-ended questions of the follow-up interview (Part B).

Item	Content	Asked in
1	“knows how”	A,B
2	“knows why”	A,B
3	“What happens in case of underdosage”	A,B
4	“What happens in case of overdosage”	A,B
5	“What to do in case of a missed dose”	A,B
6	“How long to take the OAC”	A,B
7	“Why to inform HCPs about having an OAC therapy”	A,B
8	“I would recommend this service to other patients”	B
9	“The pharmacist was able to answer all my questions”	B
10	“I am more confident about how to take my OAC”	B
11	“It is easier to take my OAC as prescribed”	B
12	“I have less concerns about my therapy with OAC”	B

**Table 2:** Characteristics of patients with vitamin K antagonist (VKA) or non-vitamin K antagonist oral anticoagulant (NOAC) therapy.

Characteristics	Total	VKA	NOAC	*p*-Value between Groups
Number of recruited patients, n (%)	81	50 (61.7%)	31 (38.3%)	-
Age in years, mean (SD)	70.7 (12.3)	69.0 (13.1)	73.3 (10.7)	ns
Gender, women (%)	34.6%	28.0%	45.2%	ns
Duration of OAC Therapy, years, mean (SD)	5.8 (5.4)	7.4 (5.7)	3.3 (3.6)	<0.01
Number of Rx-medication, mean (SD)	10.2 (4.3)	9.8 (4.5)	10.7 (4.1)	ns

**Table 3:** Number and percentages of knowledge gaps per item detected after the PMC (semi-structured interview; part A) and the follow-up interview (part B).

Item	[Part A] Semi-Structured Interview				[Part B] Follow-up Interview				before-after Comparison
	Total (n = 81)	VKA (n = 50)	NOAC (n = 31)	*p*-Value *	Total (n = 76)	VKA (n = 47)	NOAC (n = 29)	*p*-Value *	[A-B] (n = 76) *p*-Value **
1: “knows how” [n (%)]	6 (7.4)	1 (2.0)	5 (16.1)	<0.05	0	0	0	ns	n.a.
2: “knows why” [n (%)]	9 (11.1)	5 (10.0)	4 (12.9)	ns	1 (1.2)	1 (2.0)	0	ns	<0.05
3: “What happens in case of underdosage” [n (%)]	22 (27.2)	12 (24.0))	10 (32.3)	ns	4 (4.9)	2 (4.0)	2 (6.5)	ns	<0.001
4: “What happens in case of overdosage” [n (%)]	22 (27.2)	9 (18.0)	13 (41.9)	<0.05	10 (12.3)	3 (6.0)	7 (22.6)	<0.05	ns
5: “What to do in case of a missed dose” [n (%)]	51 (63)	31 (62.0)	20(64.5)	ns	15 (18.5)	11 (22.0)	4 (12.9)	ns	<0.001
6: “How long to take the OAC” [n (%)]	8 (9.9)	6 (12.0)	2 (6.5)	ns	9 (11.1)	4 (8.0)	5 (16.1)	ns	ns
7: “Why to inform HCPs about having an OAC therapy” [n (%)]	18 (22.2)	8 (16.0)	10 (32.3)	ns	2 (2.5)	2 (4.0)	0	ns	<0.005

* Chi-Square or Fisher’s exact test; ** McNemar’s test n.a. not applicable.
